# Dietary Silicon and Its Impact on Plasma Silicon Levels in the Polish Population

**DOI:** 10.3390/nu11050980

**Published:** 2019-04-29

**Authors:** Anna Prescha, Katarzyna Zabłocka-Słowińska, Halina Grajeta

**Affiliations:** Department of Food Science and Dietetics, Wroclaw Medical University, 50-556 Wrocław, Poland; katarzyna.zablocka-slowinska@umed.wroc.pl (K.Z.-S.); halina.grajeta@umed.wroc.pl (H.G.)

**Keywords:** silicon, diet, plasma, adults

## Abstract

Silicon in nutritional amounts provides benefits for bone health and cognitive function. The relationship between silicon intake from a common daily diet and silicon blood level has been scarcely elucidated, so far. The aim of this study was to analyze the associations between plasma silicon levels and the total and bioavailable silicon intake—along with the contribution of silicon made by food groups—in a healthy adult Polish population. Si intake was evaluated in 185 healthy adults (94 females and 91 males, aged 20–70) using a 3-day dietary recall and a database on the silicon content in foods, which was based on both previously published data and our own research. Fasting plasma silicon levels were measured in 126 consenting subjects, using graphite furnace atomic absorption spectrometry. The silicon intake in the Polish population differed significantly according to sex, amounting to 24.0 mg/day in women and 27.7 mg/day in men. The median plasma silicon level was 152.3 µg/L having no gender dependency but with a negative correlation with age. Significant correlations were found between plasma silicon level and total and bioavailable silicon intake, as well as water intake in the diet (*r* = 0.18, *p* = 0.044; *r* = 0.23, *p* = 0.011; *r* = 0.28, *p* = 0.002, respectively). Silicon intakes from non-alcoholic beverages, cereal foods, and carotene-rich vegetables were also positively associated with plasma silicon levels. These results may help establish dietary silicon recommendations and formulate practical advice on dietary choices to ensure an appropriate supply of silicon. The outcome of this study, however, needs to be confirmed by large-scale epidemiological investigations.

## 1. Introduction

The essentiality of silicon in human health is supported by a growing body of evidence [1,2,3]. Epidemiological studies have shown that dietary silicon was favorably related to markers of bone density and turnover. Moreover, Si in nutritional amounts may lower the risk of Alzheimer’s disease and may improve photo-damaged skin or hair and nail conditions [4,5,6,7].

The silicon level in the blood may be considered as a silicon status indicator. However, only a few reports have been published on silicon concentrations in the blood, and the values reported for fasting serum concentration in healthy adult subjects have ranged from 100 to 310 μg/L. Moreover, the relationship between silicon intake from a common daily diet and silicon blood level has so far been scarcely elucidated [8]. This results partly from the limited number of studies on the silicon content in foods and its intake in the diet.

Dietary silicon has only been assessed in certain populations so far. According to published data, a Western-type diet provides between 19 and 31 mg of Si per day [9,10]. Few studies have been performed on an appropriate dietary silicon level which would ensure beneficial health effects. An adequate intake of between 10 and 25 mg/day has been suggested, but dietary silicon levels approaching 25 mg/day or higher seem to be the most efficacious, at least for maintaining bone health in men and premenopausal women, with no adverse effects [1,5].

Although the data on silicon content in foods are incomplete, it has been assumed that cereals, along with beverages (especially beer) and some vegetables and fruits, contribute the most to the dietary intake of silicon [10,11,12]. The higher daily silicon intake of men than women which has been reported by some authors has been attributed to the typically higher beer consumption among men [9,11]. The bioavailability of silicon differs between foods: orthosilicic acid and water-soluble silicates from beverages are easily absorbed, whereas phytolytic silica, present in solid plant foods, is less absorbable [9,10,13]. The bioavailability of silicon from foods may have, along with the total silicon intake, an impact on its level in the organism.

The results of our recently published study have indicated that diet composition might be related to plasma silicon level in healthy Polish subjects [8], however no thorough analysis of this relationship has been carried out with regard to bioavailable silicon intake. The aim of this study was, therefore, to assess the relationship between plasma silicon levels and total and bioavailable silicon intake from a typical diet, with respect to the contribution of food groups into the total dietary silicon, in a healthy adult Polish population. To achieve this aim, we assessed the total silicon content in the diet, largely based on data available in the scientific literature on the silicon content of various foods. In order to complete the food silicon database used in this study, the amounts of Si contained in cereal products and beverages purchased on the Polish market were measured. The bioavailable silicon content in the diet was calculated, using previously published data on the bioavailability of this element from individual foods. The contribution of silicon from particular food groups to total silicon intake was also assessed. The plasma silicon levels in the study population were measured and then correlated with the total and bioavailable silicon contents in the diet and with the silicon intake from food groups.

## 2. Materials and Methods

### 2.1. Subjects

Two hundred and ten healthy adult subjects were recruited for the study from the Lower Silesia Regional Center of Occupational Medicine in Wrocław, Poland, from public offices and from Wrocław’s University of the 3rd Age. The exclusion criteria included serious diseases, metabolic disorders, mental health issues, and declared regular use of medication and/or dietary supplements. Of the participants that were recruited, 25 were excluded from the study because of particular dietary habits and a history of prior medication use. The study group finally consisted of 185 adults aged 20–70. All subjects gave their written informed consent. The study was conducted in accordance with the Declaration of Helsinki, and the protocol was approved by the Wrocław Medical University Ethics Board (consent No. KB-202/2012).

The anthropometric measurements and the assessment of dietary intake and habits were performed by a trained dietician for all subjects. Fasting blood samples were also drawn from 126 consenting subjects on the day of the dietary interview, and plasma samples were collected for the measurement of silicon levels—exclusively using plastic ware to prevent pre-analysis silicon contamination of the samples. Subsequent analyses were performed for the whole population and for men and women separately, considering gender differences between silicon intake and silicon blood levels, as previously reported [9,11,14]. As the silicon level in the blood has been shown to be significantly influenced by age, and because differences in silicon intake might be age-related, according to previously published studies, we decided to analyze silicon intake and serum levels in age groups largely conforming to those applied by the National Food and Nutrition Institute (Warsaw, Poland) when reporting recommended daily intake values for trace elements in adults [15]. However, groups of 66–75 year olds and >75 year olds could not be represented in our study population. Therefore the assessment of study variables was performed in the following age groups: ≤30 years, 31–50 years, and ≥51 years.

### 2.2. Dietary Intake Assessment

A 3-day dietary recall (2 weekdays and 1 weekend day in the same week) was used to assess energy and macronutrient intake, as well as silicon intake from the diet. Calculations of the composition of foods were performed using Diet v. 6.0 software with an uploaded photo album of products and meals (National Food and Nutrition Institute, Warsaw, Poland, 2019). Amounts of silicon in the diet were calculated using data from analyses of Polish food products performed at the Department of Food Science and Dietetics (Wroclaw Medical University) and from published data on the silicon content of foods originating in Poland, the UK, Belgium, the USA, and South Korea [9,10,16,17,18,19,20,21]. The following products purchased on the Polish market were analyzed in our laboratory: cereal products, fruit and vegetable juices, and mineral and spring waters. The resulting data on the silicon content of these products were then included into the food silicon database created using previously published data. For multiple data reported on the same foods, the following order of priority was applied: (1) the reliability of the method used for silicon measurement, (2) the degree of representativeness of the data, and (3) the origin of the products analyzed.

Bioavailable silicon in the diet was estimated using published data from human research on excreted silicon after the consumption of various foods with a determined silicon content [9]. In several cases, data on the bioavailability assessed by in vitro experiments were also used [10]. Bioavailable silicon was calculated for foods providing 93.9% ± 3.8% of the total silicon in the diet of the study group, and the percentage of bioavailable silicon in these foods was 36.5% ± 4.0%. This value was used for foods when no data on their bioavailability was available.

In addition to total and bioavailable silicon intake from the diet, the contribution of silicon from particular food groups to the total amount in the diet was assessed and expressed in percentage of total silicon intake. The database used for total silicon intake calculation was also applied for the calculation of silicon in food groups. The silicon intakes from individual foods were added up to obtain the main food groups as well as subgroups of foods relevant in silicon intake. For example, silicon intake from cereal products was calculated in the following groups: refined grain foods, comprising white breads and rolls, foods made from white flour and white flour pasta, whole grain foods comprising wholemeal and wholegrain breads and rolls, cereal flakes, cereal snacks, groats, and wholemeal dishes.

### 2.3. Food Sampling for Silicon Analysis

Cereal food products: breads, groats, flakes, rice, pasta, and beverages in cartons and plastic bottles, along with bottled mineral and spring waters, were purchased in domestic grocery stores and hypermarkets (Lower Silesia region). In the case of beverages, at least three production batches of each product were collected at least 1 month apart. In total, 486 food products from 99 manufacturers were analyzed for silicon. Solid food samples were finely ground in a mill, then packed in polyethylene bags and stored at room temperature prior to analysis. The samples of bread were dried in a laboratory oven and the dry weight was measured. The juice samples were stored in plastic flasks below −20 °C prior to analysis. Water samples for analysis were taken directly from the original bottles stored at room temperature.

### 2.4. Silicon Measurement in Foods

The solid food and juice samples were thoroughly blended and then mineralized (0.5 g) with 4 mL 65% HNO_3_ (Instra-Analyzed JTBaker, USA) and 1 mL 30% H_2_O_2_ (Ultrex II JTBaker, USA) in a microwave oven (Milestone 1200 Mega, USA). The mineralization was done according to the manufacturer’s recommendations. The blank digests were carried out in the same way. Measurements of the silicon content in the mineralized samples and the bottled water samples were performed on a Perkin Elmer PinAAcle 900 (USA) atomic absorption spectrometer in graphite furnace mode (GF-AAS). The operating parameters of the GF-AAS for the silicon analysis are presented in Supplementary Table S1. Two samples of each food product were measured four times each. The plastic and Teflon utensils were pre-cleaned or rinsed in 10% HNO_3_ and then rinsed in deionized water. All reagents were prepared using deionized water, with a specific resistivity of 18.2 MΩ cm. The calibration working standard solutions were prepared from a silicon standard solution of 1 mg/L, as (NH_4_)_2_SiF_6_ in H_2_O (Perkin Elmer, USA), in a silicon concentration ranging from 40 to 120 μg/L. The samples were measured directly, or after appropriate dilution with deionized water. The mean recovery of total silicon obtained for selected spiked samples of food was 101.2%. Accuracy and precision were also assessed by measuring the silicon in certified reference materials: NCS ZC73008 Rice (NACIS, China) and MISSIPPI-03 River Water (Environment Canada, Canada). The agreement with the defined values was 95.2% and 100.5%, respectively.

The resulting data on the silicon content of cereal products and beverages were then included in the food silicon database which was used to assess the silicon intake of the healthy Polish subjects. Data on Si in cereal products that require preparation with water were re-calculated, taking into account the final percentage of water.

### 2.5. Silicon Measurement in Plasma

The direct measurement of silicon in plasma was performed by GF-AAS (PinAAcle 900, Perkin Elmer, USA) using the method of standard addition calibration described elsewhere [8]. The operating conditions and instrumental parameters for silicon measurement are summarized in Supplementary Table S1.

### 2.6. Statistical Analyses

Statistical calculations were performed using Statistica StatSoft 13.0. The distribution of continuous variables (dietary intake, food consumption, and plasma silicon concentration) was checked using the Shapiro–Wilk test. Depending on the distribution of variables, either Student’s *t*-test or the Mann–Whitney U test were used for gender comparisons (dietary intake, food group consumption and plasma silicon concentration); either Tukey’s HDS test or the Kruskal–Wallis test was used for age comparisons (dietary intakes, food group consumption, and plasma silicon concentration). Pearson correlation analysis was used to measure the association between plasma silicon and age, and age-adjusted partial correlation analyses were performed for the determination of associations between plasma silicon level and the dietary variables. The correlation analyses were carried out in the whole study population and in both gender groups. Pearson’s χ^2^ test was used to assess any differences in age distribution between the gender groups.

## 3. Results

### 3.1. Study Population Characteristics, Dietary Intake, and Dietary Habit Assessment

The baseline characteristics of the subjects, as well as the dietary intake of energy and macronutrients are presented in Table 1.

### 3.2. Silicon Content in Cereal Products and Beverages from the Polish Market

The determination of silicon was performed in cereal products that are generally of local origin in stores, and for which no data on silicon content were available. Silicon content was also measured in beverages, which are mostly manufactured by domestic producers usually from concentrates reconstituted with water. The content of silicon in cereal products on the Polish market varied over a wide range, from 0.94 mg/100 g in Basmati rice to 14.03 mg/100 g in oat bran (Table 2). Refined products contained less silicon than wholemeal products and wholegrain products. There were also differences between grain species, with the highest values found in oat, millet, and barley products. High amounts of silicon were also found in unrefined rice products. The content of silicon in selected beverages—juices and bottled water available on the Polish market—ranged between 0.63 and 1.22 mg/100 (Table 3). The fruit juices we analyzed may provide silicon in amounts similar to vegetable and fruit–vegetable juices. Mineral waters contained 25% more Si on average than spring waters. As the silicon content in beverages was measured in at least three production batches of products, the coefficient of variance (CV) was calculated for each product, in order to assess the variability of silicon levels in beverages available on the market. The highest CV values were obtained for orange juices (Table 3).

### 3.3. Silicon Intake

#### Total and Bioavailable Silicon in the Diet and Silicon Intake from Food Groups

The total intake of silicon in the study group, the amount and percentage of bioavailable silicon, and the contribution of silicon from particular food groups to total silicon intake are presented in Table 4. It may be noted that men consumed significantly more silicon in their diet than women, however, no differences in total silicon intake were found between the age groups of the study population. Bioavailable silicon comprised 36.5% of total dietary silicon in the study population. In line with total silicon, both the bioavailable silicon content and its percentage in total silicon were higher in men than in women. A higher percentage of bioavailable silicon in the diet was also shown for the younger subjects rather than the older ones. Non-alcoholic beverages in total were found to be a major source of silicon in the diet. These products provided ca. 30% of dietary silicon in the study population, with an equivalent contribution made by hot beverages (tea and coffee) and cold ones. However, women gained more silicon from hot beverages than men, and in the youngest subjects a higher intake of silicon from cold non-alcoholic beverages was noticed in comparison with older age groups. The high contribution of cereal products in total, accounting for 25.8% of the amount of silicon in the diet, was also revealed. Among them, whole grain foods were shown to be more important in providing silicon for women than for men. Fruit and vegetables provided considerable amounts of silicon, whilst the lowest contribution of these products to silicon intake was shown for subjects aged ≤30. Divergent eating patterns influenced the contribution of silicon from meats, eggs, and fats to the total silicon intake in both sexes, as these products provided almost half as much silicon in the diet of women than of men.

### 3.4. Plasma Silicon and Its Relation to Diet

Fasting plasma silicon levels were measured in 126 of the study participants; the median value amounted to 152.3 μg/L, with no significant differences in terms of gender (Table 4). However, an impact of age on plasma silicon level was noted. Subjects younger than 31 years had higher silicon plasma levels than those in the older age groups. This was confirmed by the negative correlation of plasma silicon values with age (r = −0.40, *p* <0.000). Further correlation analyses were performed after adjusting for age and measured anthropometric parameters (body mass index and body weight). In the assessment of dietary impact on plasma silicon, we included the following dietary variables: total and bioavailable silicon intake, macronutrient intakes, and silicon intakes from food groups and subgroups. Animal protein-rich foods (meat and meats products, fish and fish eggs, and milk products) were taken into consideration in total, as the intake of silicon from these products was relatively low. Only the significant correlations are presented in Table 5.

Plasma silicon levels positively correlated with total and bioavailable silicon content in the diet—along with water intake—in the study population. Moreover, a negative correlation with fat and animal protein was noted. The silicon intake from particular food groups was also associated with plasma silicon level. Among them, the consumption of cold, non-alcoholic beverages was shown to be positively related to silicon concentration in the blood. Silicon intake from cereal products in total was not associated with plasma silicon level, although positive correlations were found for selected cereal products. From among vegetable and fruit food groups, a positive correlation with plasma silicon was recorded only for carotene-rich vegetables. A negative relationship found for silicon from animal protein-rich foods supported the negative correlation for animal protein intake.

## 4. Discussion

In our study, we assessed thoroughly the relationship between silicon intake from a typical diet and fasting plasma silicon level, taking into consideration both total dietary silicon and bioavailable silicon, as well as silicon intake from food groups.

In the assessment of dietary silicon in the Polish population we found it necessary to complete the database on the silicon content of foods, by determining the silicon content of cereals and non-alcoholic beverages available on the Polish market. Cereals greatly contribute to the energy intake of the Polish population, according to the National Multicenter Health Survey (WOBASZ II, 2013–2014), and may provide considerable amounts of silicon in the diet [16,22]. The silicon content of these products on the Polish market, however, had not been measured. Beverages were shown to be important sources of highly absorbable orthosilicic acid [9,10], but there was only a single study published on the silicon content of these products in Poland [19]. Our results confirmed the high silicon content of cereal products found by other authors, though some differences were shown, including a lower silicon content of Polish wholemeal and wholegrain breads [16,17]. In the fruit and vegetable juices analyzed in our study, a lower range of silicon concentration was recorded than the range previously published for Polish products (0.12–2.67 mg/100 g) [23]. This discrepancy might result from the relatively high variation of silicon content in these products, even among different production batches of the same brand. As the juices were not fortified, the differences might result from the variable content of silicon in the raw material or in the water used in the manufacturing process. This indicates that the contribution of juices to silicon intake may vary even if the same product is consistently present in the diet. In the mineral waters and spring waters analyzed, we found higher silicon concentrations than those reported for bottled waters produced in northern Poland [19]. The differences in silicon content might therefore be attributed to the geographic features of the area from which the water is sourced [24].

Self-selected food consumption in the Polish population has translated into a daily intake of silicon which is close to the lower limit (25 mg/day) that is considered beneficial. This finding was also similar to the results obtained from the original Framingham and Framingham Offspring cohorts [9]. Nevertheless, the negative impact of age on total silicon intake found in the Framingham study was not confirmed in our study group. The higher amount of silicon in men observed in our study might not be attributed to higher beer consumption in this gender group—as previously reported [9,10,11]—because the overall consumption of alcohol in men was low and did not differ significantly from that of women. The distribution of foods contributing to silicon intake in our study population confirmed the significant role of cereals and non-alcoholic beverages in the provision of silicon, and the smaller but still relevant position of fruits and vegetables in silicon intake, as shown elsewhere [9,10,11].

In our assessment of dietary silicon, we also calculated the proportion of total intake of Si which represented a bioavailable form of silicon. As the data used in these calculations originated from experiments estimating silicon absorption from individual foods [9,10,13], the dietary silicon bioavailability presented in this study should be regarded as the potential bioavailability from the diet not taking account the factors that can influence the uptake of silicon from mixed meals. Some gender and age-related divergences in dietary habits, and therefore in food contributions to total silicon intake, have corresponded with differences in bioavailable silicon amount in the diet. The higher contribution of cold non-alcoholic beverages to silicon intake in subjects aged under 31 translated into a higher percentage of bioavailable silicon in their diet. Meat products—providing more silicon in the diet of male subjects than female ones—were shown to contain available silicon amounting to more than 70% of total silicon [10]. Moreover, despite the low consumption of alcoholic beverages in the study population, men tended to consume more of the products that are known for their high bioavailable silicon content, especially beers. This pattern of consumption resulted in a higher intake of bioavailable silicon in this gender group. The actual bioavailability of silicon from a mixed diet needs to be investigated in order to collect more reliable data.

Silicon content in the diet of the study population was then tested against plasma silicon level. As with results reported for the German population, plasma silicon showed an inverse relationship to age [14]. After adjusting for age and anthropometric parameters, the total silicon content in the diet of healthy adults was shown to be weakly correlated with plasma silicon level. To date, only one study—of an adult Korean population—has investigated the associations between self-selected food consumption and body silicon status, determined by silicon urine excretion [11]. The authors reported that dietary silicon, assessed by the food record method and their own food silicon database, was significantly positively related to diurnal silicon in the urine among males alone. When taking into consideration bioavailable dietary silicon, its relationship to plasma silicon level was slightly more pronounced in the Polish population, indicating that the consumption of a diet rich in bioavailable silicon may be important for the maintenance of body silicon status. The positive relationship between plasma silicon level and silicon intake from non-alcoholic beverages, which was accompanied by a positive correlation with water intake, clearly confirms the substantial role of foods rich in monomeric silicon, such as spring water, mineral water, and fruit and vegetable juices, in providing silicon to the human body [10,13]. This association was not observed for hot beverages (coffee and tea), which may be related to coffee and tea polyphenols affecting the availability of minerals [25]. However, in our recent study we noticed a positive association between plasma silicon and tea and coffee consumption in rheumatoid arthritis patients [8]. As the body silicon status in rheumatoid arthritis seems to be affected, other factors related to the disease may interfere with the associations found. A significant correlation with plasma silicon was also observed for some cereal foods, including groats, which are known to be generally rich in silicon (especially barley and millet, see Table 2). Taking into consideration the high contribution of cereal products to the total silicon intake in Polish populations and in others, their role as a source of dietary silicon and in maintaining silicon levels in the body appears to be significant. Among carotene-rich vegetables, which also positively correlated with plasma silicon, green beans and spinach have been reported to be high-silicon foods [16,23]. This might have an impact on the observed correlation. Despite the high bioavailability of silicon from animal foods, their relationship with plasma silicon level was negative, as in the case of silicon from fats. The influence of the chemical environment created by food components on the absorption and retention of silicon has not yet been recognized, however, the impact of dietary fat on the absorption of trace elements could play a role, among others [26].

The results of this study may be helpful in establishing dietary recommendations on silicon and the formulation of practical advice on dietary choices to ensure its appropriate supply. Further studies are merited in order to explain the mechanisms which interfere with Si absorption and retention in the body.

## 5. Conclusions

In our study, significant correlations were found between plasma silicon level and the total and bioavailable silicon intake, and the consumption of fluids, cereal foods, and carotene-rich vegetables. However, our findings need to be supported by large-scale epidemiological studies. Moreover, a number of issues concerning dietary silicon interactions with nutrients and metabolic factors must still be elucidated.

## Figures and Tables

**Table 1:** Characteristics of the study population and dietary intake.

	All Subjects (*n* = 185)	Female (*n* = 94)	Male (*n* = 91)
**Characteristics**			
Age (years), median (range)	45.1 (20.2–70.2)	45.2 (23.6–68.2) ^a^	45.1 (20.2–70.2) ^a^
≤30 years, *n* (%)	49 (26.5)	23 (24.5) ^A^	26 (28.6) ^A^
31–50 years, *n* (%)	74 (40.0)	38 (40.4)	36 (39.7)
≥51 years, *n* (%)	62 (33.5)	33 (35.1)	29 (31.9)
BMI (kg/m2), median (range)	25.6 (18.3–32.0)	24.4 (18.3–32.0) ^a^	25.9 (18.8–31.8) ^b^
Body weight (kg), median (range)	73.0 (46.5–130.0)	63.0 (46.5–86.0) ^a^	78.0 (60.0–130.0) ^b^
**Dietary intake, median (Q1–Q3)**			
Energy (MJ/day)	8.07 (7.19−9.42)	7.57 (6.97−8.12) ^a^	9.35 (8.12−10.70) ^b^
Nutrients (g/day)			
Protein	76.5 (63.1–92.7)	66.8 (57.9–78.2) ^a^	91.3 (74.0–108.2) ^b^
Carbohydrates	281.4 (246.1−319.6)	269.3 (236.6−300.5) ^a^	304.1 (254.6−350.1) ^b^
Fat	58.2 (42.8−77.7)	46.1 (36.6−59.7) ^a^	72.7 (54.8−90.7) ^b^
Ash	17.7 (14.8−21.3)	16.3 (15.9−13.4−18.6) ^a^	20.3 (17.5−23.8) ^b^
Fiber	21.9 (17.6−27.7)	21.6 (16.3−25.6) ^a^	22.9 (18.3−30.4) ^b^
Water	2158 (1715−2634)	2053 (1626−2589) ^a^	2269 (1886−2686) ^b^
Alcohol	3.0 (0.0–6.4)	0.0 (0.0–5.0) ^a^	6.0 (0.0–10.7) ^a^

BMI—body mass index; Q1–Q3—range between 25th and 75th percentiles. Values in the same row which do not share the same superscript letter are significantly different (*p* <0.05). For the variable comparison between female and male groups, lowercase letters were used; for the comparison of age-group distribution in the female and male groups, uppercase letters were used.

**Table 2:** Silicon content in cereal products (mg/100 g).

Product	n	Mean ± SD
**Breads**		
Bread, white	15	1.88 ± 0.83
Rolls, white	10	1.59 ± 0.64
Breads, wholemeal & wholegrain	13	2.00 ± 0.63
Rolls, wholemeal & wholegrain	6	1.82 ± 0.48
Crispbread	15	3.97 ± 3.62
Matzo, classic	2	1.50 ± 0.12
Matzo, wholemeal	2	2.45 ± 0.13
**Groats**		
Couscous	4	2.35 ± 0.78
Buckwheat & Roasted buckwheat	9	1.17 ± 0.59
Millet	5	7.96 ± 0.71
Barley	6	6.64 ± 3.73
Corn	3	1.67 ± 1.71
**Flakes**		
Corn	5	2.12 ± 0.46
Oat	5	13.89 ± 2.62
Wheat	3	2.49 ± 0.59
Spelt	4	2.42 ± 0.87
Rye	3	2.29 ± 0.40
Muesli (various types)	6	4.58 ± 2.31
**Bran**		
Oat	3	14.03 ± 7.69
Wheat	4	6.80 ± 2.19
**Rice**		
Long grain, white	4	3.41 ± 2.62
Jasmine, white	4	1.47 ± 0.28
Basmati, white	4	0.94 ± 0.30
Whole grain, brown	10	9.72 ± 2.57
Chinese, black	3	10.63 ± 7.46
**Wild rice** (*Zizania aquatica*)	4	2.99 ± 0.44
**Pasta**		
Wheat, white	4	1.22 ± 0.31
Wheat, wholemeal	5	5.20 ± 2.82
Spelt	2	2.86 ± 0.64
Rye	2	5.28 ± 1.24
Mixed grain	2	5.63 ± 1.41

SD—standard deviation.

**Table 3:** Silicon content in beverages (mg/100 g).

Product	*n*	Mean ± SD	Range of CV Values for Products from a Single Brand (%) *
**Juice**			
Orange	8	0.87 ± 0.35	32–99
Apple	8	0.90 ± 0.58	7–33
Grapefruit	8	0.63 ± 0.39	11–54
Blackcurrant	3	0.66 ± 0.05	13–32
Multi-fruit	4	1.22 ± 0.29	14–28
Carrot–Fruit	6	1.17 ± 0.48	16–57
Tomato	6	0.75 ± 0.22	11–38
Multi-vegetable	7	0.76 ± 0.26	6–34
**Bottled water**			
Spring	5	0.77 ± 0.40	3–42
Mineral	17	0.96 ± 0.63	3–50

SD—standard deviation; CV—coefficient of variation, %. * CV assessed by analysis of at least 3 samples, from different production batches, of each shop-bought product.

**Table 4:** Total silicon intake, contribution of food groups to silicon intake, bioavailable Si in the diet, and plasma silicon, median (Q1–Q3).

	All Subjects	Female	Male	Age Groups (Years)		
				≤30	31–50	≥51
Silicon Intake (mg/day)	26.1 (22.0−30.3)	24.0 (21.2−29.7) ^a^	27.7 (23.4−31.6) ^b^	25.2 (22.7−29.0) ^α^	26.0 (21.5−30.2) ^α^	26.4 (22.7−31.9) ^α^
**Silicon Intake from Food Groups (% Of Total Si intake)**						
Refined grain foods	17.9 (12.2−23.9)	19.3 (12.4−24.7) ^a^	17.2 (11.9−21.5) ^a^	18.3 (13.6−24.6) ^α^	18.0 (11.5−24.4) ^α^	17.5 (12.2−20.2) ^α^
Whole grain foods	7.9 (1.3−13.0)	10.0 (4.0−14.2) ^a^	4.6 (0.0−11.2) ^b^	5.5 (1.0−10.8) ^α^	5.8 (1.5−13.0) ^α^	10.5 (2.6−14.1) ^α^
Potatoes and Starches	2.5 (1.3−4.0)	2.3 (1.1−3.3) ^a^	3.0 (1.8−5.2) ^b^	2.4 (1.2−3.4) ^α^	2.5 (1.5−4.0) ^α^	2.6 (1.4−4.5) ^α^
Vegetables	8.5 (5.9−11.7)	8.3 (5.6−11.6) ^a^	8.7 (6.4−11.9) ^a^	7.4 (5.2−10.7) ^α^	8.9 (7.0−12.9) ^β^	8.7 (5.9−12.0) ^αβ^
Fruits	10.4 (5.5−15.0)	11.1 (5.6−15.1) ^a^	9.6 (5.5−14.4) ^a^	6.4 (2.5−12.8) ^α^	11.0 (6.0−15.2) ^αβ^	11.8 (7.8−15.0) ^β^
Cold, n/a beverages	15.1 (7.1−24.1)	14.8 (7.3−25.7) ^a^	15.2 (6.9−23.6) ^a^	21.9 (13.6−30.5) ^α^	11.1 (6.8−19.9) ^β^	16.7 (5.4−22.2) ^αβ^
Tea & Coffee	15.0 (9.9−21.2)	17.4 (11.5−22.5) ^a^	14.0 (9.1−19.8) ^b^	14.3 (9.3−20.6) ^α^	15.2 (10.3−21.0) ^α^	14.4 (8.7−21.0) ^α^
Dairy products	1.7 (0.9−2.9)	1.7 (1.0−2.8) ^a^	1.8 (0.9−2.9) ^a^	1.9 (1.1−3.0) ^α^	1.9 (1.2−3.2) ^α^	1.1 (0.8−2.3) ^α^
Meats & Meat products	4.8 (2.3−9.2)	3.9 (1.8−7.8) ^a^	6.5 (2.7−11.2) ^b^	4.8 (1.8−8.1) ^α^	5.1 (2.9−10.3) ^α^	4.5 (2.1−9.2) ^α^
Fish & Fish products	0.2 (0.0−1.0)	0.2 (0.0−0.8) ^a^	0.4 (0.0−1.3) ^a^	0.2 (0.0–1.7) ^α^	0.0 (0.0–0.5) ^α^	0.4 (0.0–1.5) ^α^
Eggs	1.0 (0.3−2.1)	0.8 (0.3−1.6) ^a^	1.3 (0.3−2.4) ^b^	0.6 (0.1–1.5) ^α^	1.3 (0.6–2.2) ^α^	0.9 (0.3–2.0) ^α^
Fats	1.3 (0.7−2.1)	1.1 (0.6−2.0) ^a^	1.5 (0.9−2.3) ^b^	1.3 (0.8−2.1) ^α^	1.3 (0.8−2.3) ^α^	1.4 (0.7−2.0) ^α^
Legumes	0.2 (0.0−1.1)	0.3 (0.0−0.5) ^a^	1.0 (0.0−1.3) ^a^	0.2 (0.0–0.7) ^α^	0.5 (0.0–1.5) ^α^	0.2 (0.0–0.9) ^α^
Nuts & Seeds	0.4 (0.0−0.3)	0.3 (0.0−0.4) ^a^	0.5 (0.0−0.2) ^a^	0.6 (0.0–1.0) ^α^	0.4 (0.0–0.6) ^α^	0.4 (0.0–0.9) ^α^
Sugar & Sweets	0.3 (0.1−0.7)	0.3 (0.1−0.5) ^a^	0.4 (0.1−0.9) ^a^	0.4 (0.2–0.9) ^α^	0.3 (0.1−0.6) ^α^	0.3 (0.1−0.6) ^α^
Alcoholic beverages	1.8 (0.0−3.7)	0.0 (0.0–2.9) ^a^	4.1 (0.0–6.3) ^a^	2.2 (0.0–6.6) ^α^	2.0 (0.0–4.2) ^α^	0.0 (0.0–4.4) ^α^
Others	0.9 (0.6−1.2)	0.9 (0.6−1.1) ^a^	1.0 (0.7−1.3) ^a^	0.8 (0.6−1.1) ^α^	0.9 (0.7−1.2) ^α^	0.9 (0.6−1.3) ^α^
**Bioavailable Dietary Silicon**						
(mg/day)	9.4 (7.7−11.5)	8.6 (7.1−11.0) ^a^	9.9 (5.5–29.6) ^b^	9.7 (8.0−11.0) ^α^	9.0 (7.4−11.5) ^α^	9.7 (8.0−11.9) ^α^
% total silicon in diet	36.5 (33.6−38.9)	36.2 (33.4−38.5) ^a^	37.2 (28.5–47.9) ^b^	37.5 (35.9−39.5) ^α^	36.2 (33.2−38.7) ^β^	36.4 (33.4−38.8) ^β^
**Plasma Silicon**						
(μg/L)	152.3 (116.3−195.6)	168.5 (121.2−208.2) ^a^	144.8 (114.7−175.0) ^a^	197.8 (140.5−224.1) ^α^	147.9 (120.8−171.6) ^β^	123.6 (94.2−180.1) ^β^
*(n)*	*−126*	*−64*	*−63*	*−32*	*−46*	*−48*

Q1–Q3—range between 25th and 75th percentiles; n/a—non-alcoholic. Values in the same row which do not share the same superscript letter are significantly different. For variable comparisons between female and male groups, Latin lowercase letters were used; for comparisons between age groups, Greek lowercase letters were used.

**Table 5:** Significant partial correlations between plasma silicon level and dietary variables (*n* = 126).

Variable	r ^†^	*p*-Value
**Nutrient (intake/day)**		
Silicon	0.18	0.044
Bioavailable silicon	0.23	0.011
Water	0.28	0.002
Fat	−0.19	0.036
Animal protein	−0.18	0.045
**Silicon from food groups and subgroups (intake/day)**		
Cold, non-alcoholic beverages	0.21	0.022
Mineral & Spring water	0.22	0.013
Fruit & Vegetable juices	0.22	0.011
Groats	0.19	0.037 *
White rice	0.2	0.034
Cereal flakes	0.32	0.009 **
Carotene-rich vegetables	0.28	0.002
Animal protein-rich foods	−0.18	0.045

^†^ Partial correlation coefficient, adjusted for age, body mass index and body weight. * only in female subjects. ** only in male subjects.

## Supplementary Materials

The following are available online at https://www.mdpi.com/2072-6643/11/5/980/s1, Table S1: The operating conditions and instrumental parameters for silicon determination in food and plasma samples by graphite furnace atomic absorption spectrometry.
