# Supplementary material for: Dietary Silicon and Its Impact on Plasma Silicon Levels in the Polish Population
Source: Nutrients. 2019 Apr 29;11(5):980. doi: 10.3390/nu11050980 (PMC6567281; doi:10.3390/nu11050980)
Supplement: Supplementary file 1 [file nutrients-11-00980-s001.pdf]

Supplementary Table S1. The operating conditions and instrumental parameters for silicon determination in food and plasma samples by graphite furnace atomic absorption spectrometry (GF-AAS).

**Operating conditions**	**Temperature (°C)**	**Ramp time (s)**	**Hold time (s)**	**Gas**
**Food samples**				
Dry	110	1	30	Argon
	130	15	50	Argon
Pyrolyze	1250	10	20	Argon
Atomize	2450	0	5	-
Clean	2550	1	3	Argon
**Plasma samples**				
Dry	110	1	5	Argon
	130	30	40	Argon
	300	20	5	Argon
Mineralize	550	20	15	Air
	550	1	15	Argon
Pyrolyze	1100	5	10	Argon
	1250	1	30	Argon
Atomize	2450	0	5	-
Clean	2550	1	3	Argon
Instrumental parameters				
Wavelength		251.61 nm		
Slit width		0.2 nm		
Lamp energy		63 mA		
Measurement mode		Area under the peak		
Characteristic mass		42 pg		
Gas flow		250 mL/min		
